# Supplementary material for: MALDI Imaging, a Powerful Multiplex Approach to Decipher Intratumoral Heterogeneity: Combined Hepato-Cholangiocarcinomas as Proof of Concept
Source: Cancers (Basel). 2023 Apr 4;15(7):2143. doi: 10.3390/cancers15072143 (PMC10093162; doi:10.3390/cancers15072143)
Supplement: Supplementary file 1 [file cancers-15-02143-s001.zip › cancers-2296979-supplementary.pdf]

cancers

MDPI

Supplementary materials

Supplementary Table S1: Immunohistochemistry list

Anticorps anti-	Dilution	Clonality	Supplier	Reference
CK7	1/500	50V-TL 12/30	DAKO	M7018
CK19	1/200	RCK108	DAKO	M0888
Glypican-3	1/100	1G12	Zytomed	MSK067-05
EpCam Ber-EP4	1/50	Ber-EP4	DAKO	M0804
Nestin	1/500	10c2	Santa Cruz	sc-23927

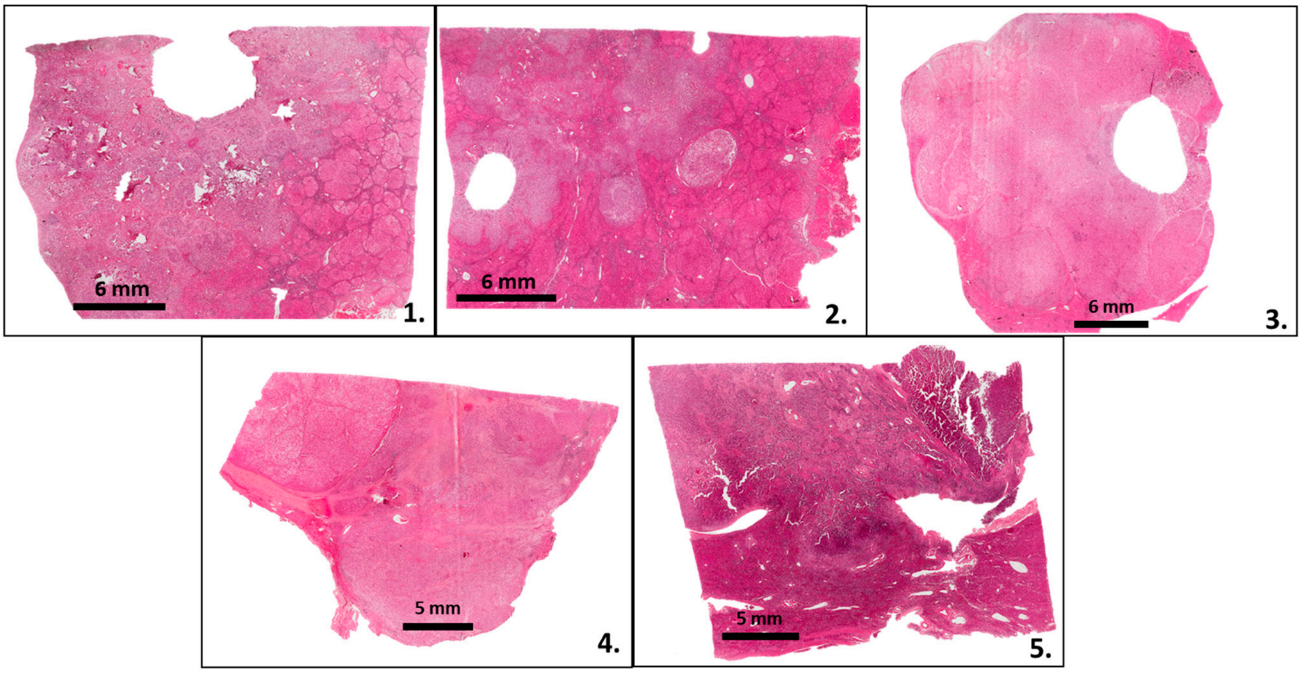

Supplementary Figure S1: Hematein Eosin Safran (HES) staining of the five cases.

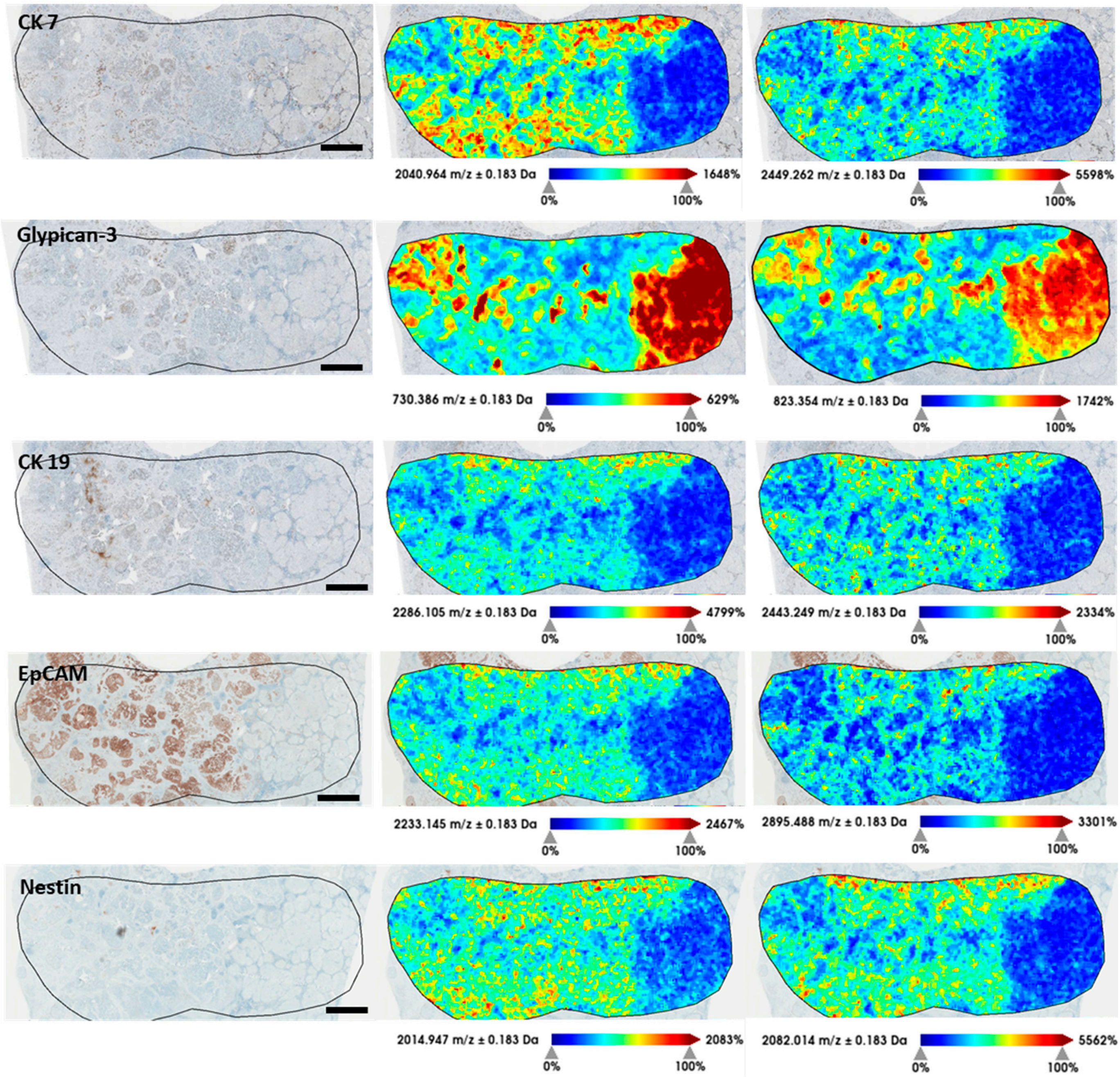

Supplementary Figure S2: Comparison of a selection of tryptic peptides derived from current IHC markers and the corresponding IHC for the patient 1. Scale bar = 3 mm.

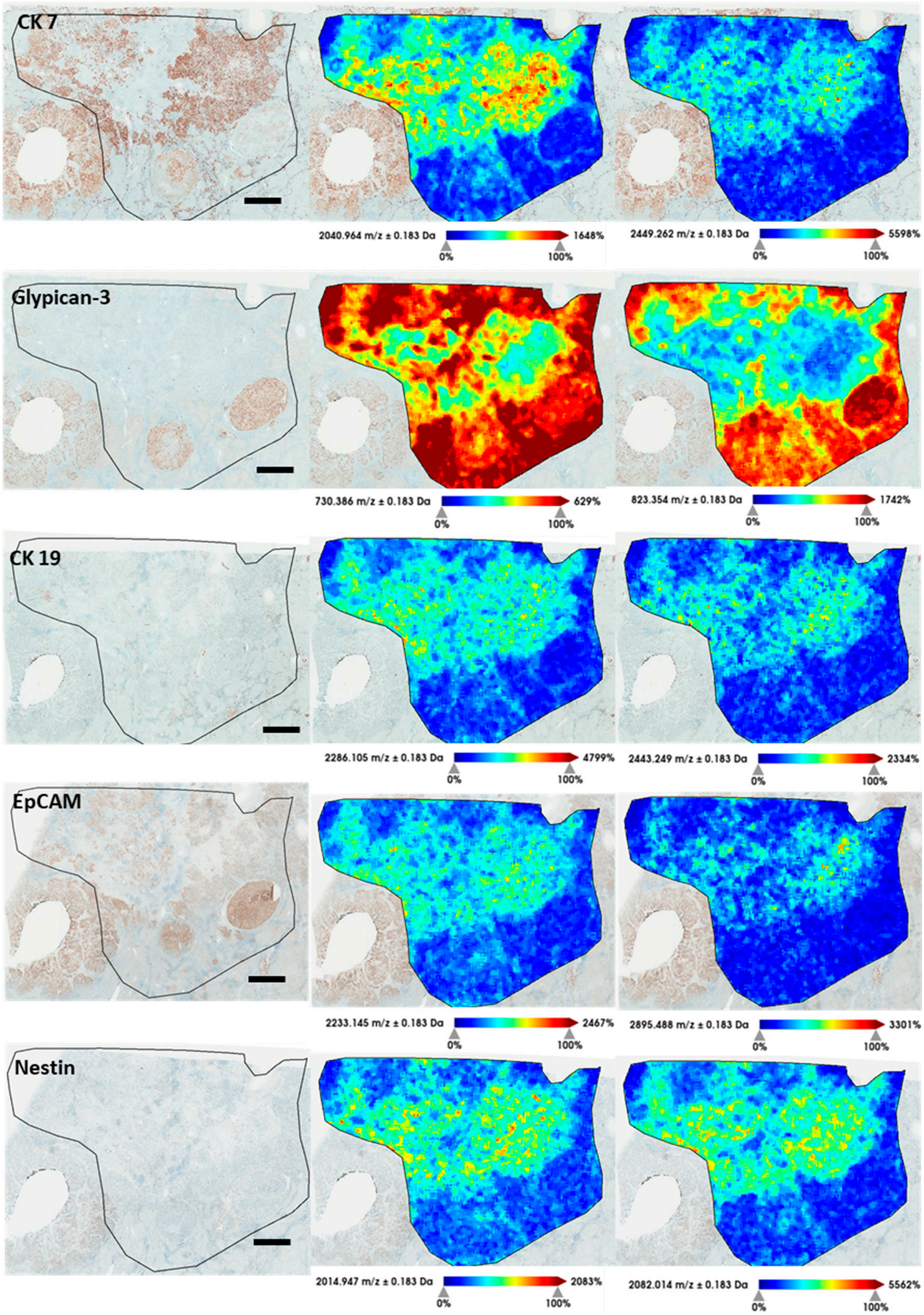

Supplementary Figure S3: Comparison of a selection of tryptic peptides derived from current IHC markers and the corresponding IHC for the patient 2. Scale bar = 2 mm.

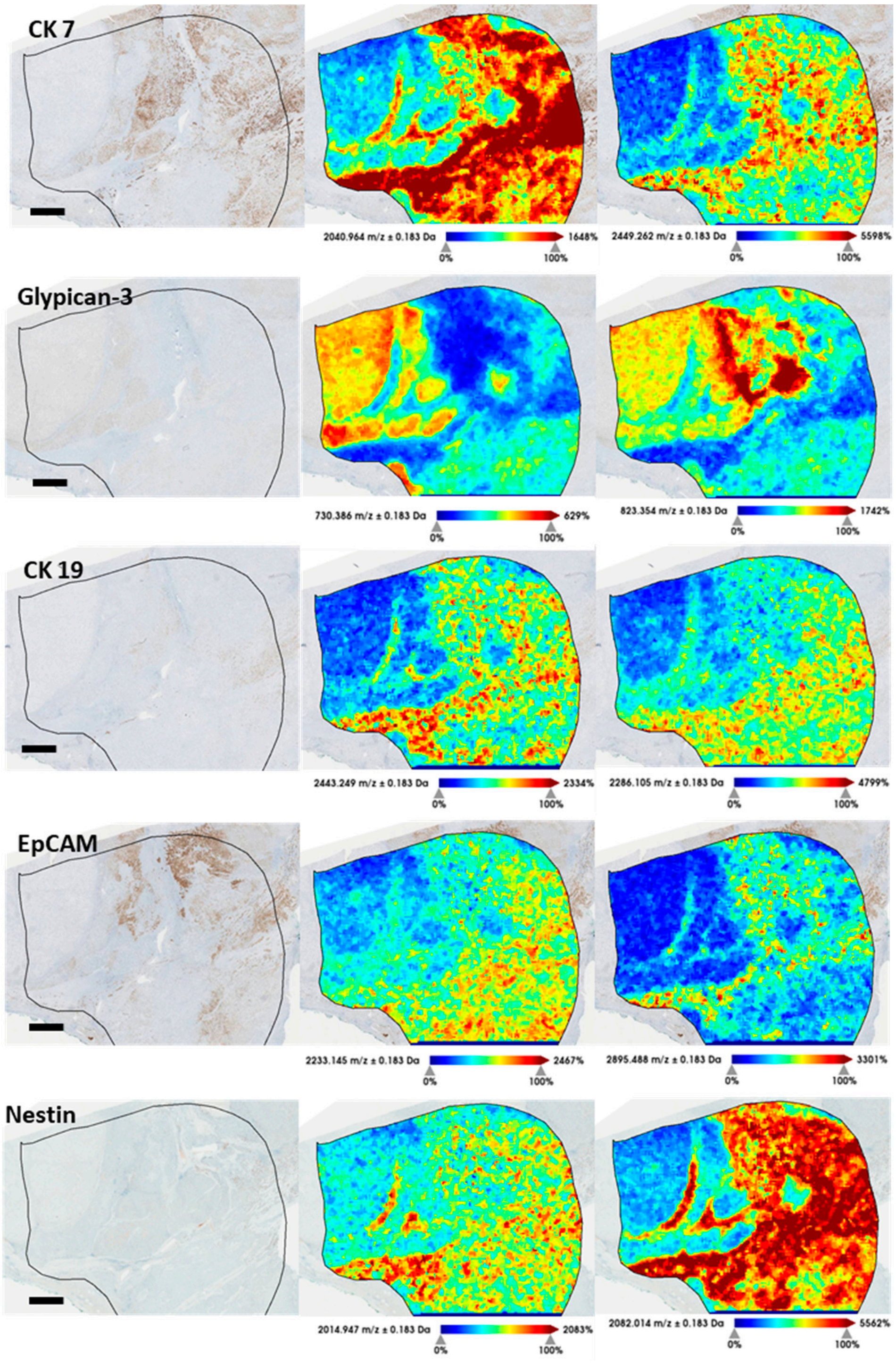

Supplementary Figure S4: Comparison of a selection of tryptic peptides derived from current IHC markers and the corresponding IHC for the patient 4. Scale bar = 2 mm.

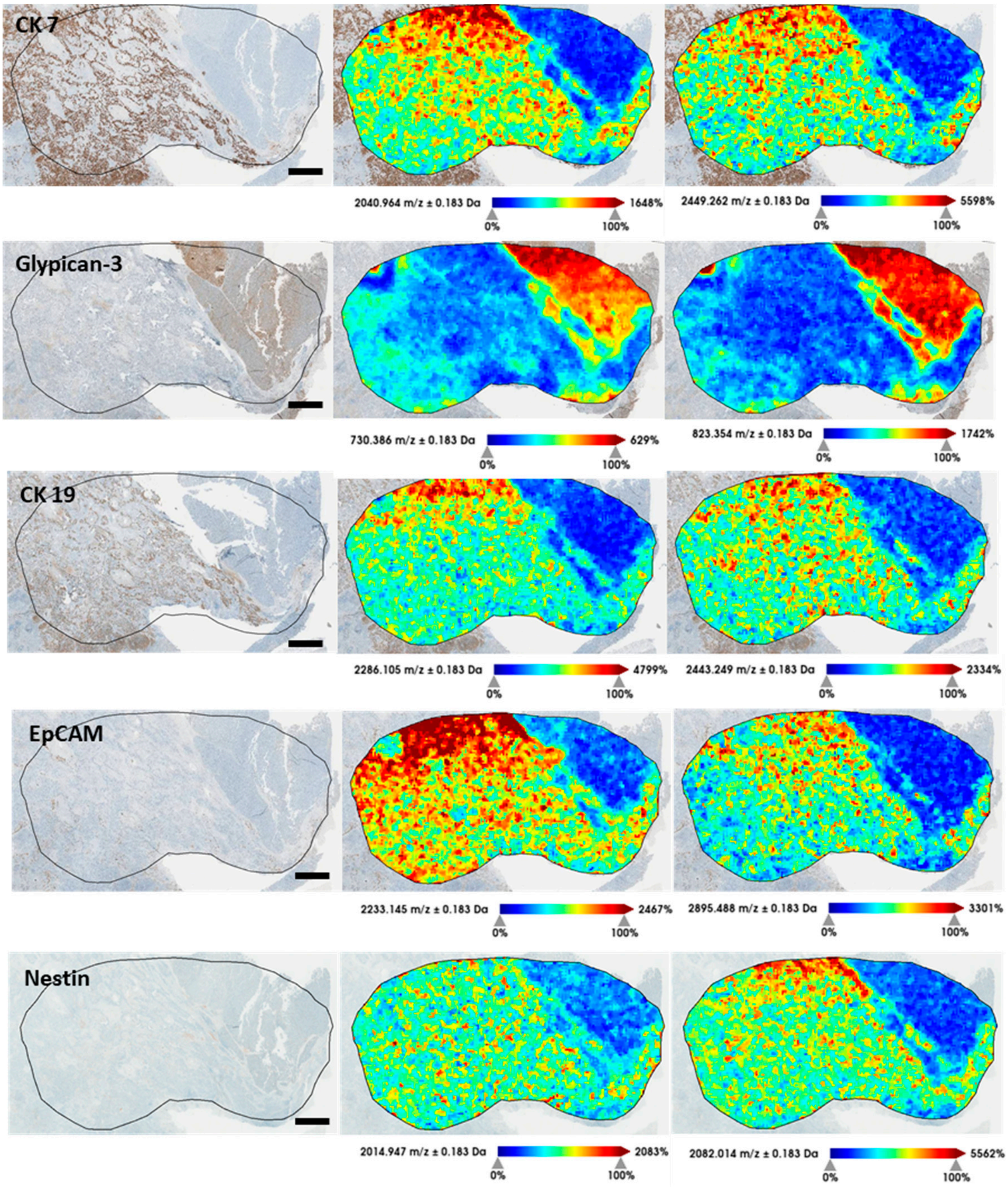

Supplementary Figure S5: Comparison of a selection of tryptic peptides derived from current IHC markers and the corresponding IHC for the patient 5. Scale bar = 2 mm.

Supplementary Table S2: in silico digested peptides

	**Sequences**	**theo m/z**	**exp m/z**	**delta**
**CK7**	EVTINQSLLAPLRLDADPSLQR	2449,3409	2449,262	0,0789
	SIHFSSPVFTSRSAAFSGR	2041,025	2040,964	0,061
**CK19**	MTSYSYRQSSATSSFGGLGGGSVR	2443,1306	2443,249	-0,1184
	EVAGHTEQLQMSRSEVTDLR	2286,1142	2286,105	0,0092
**EpCam**	AKPEGALQNNDGLYDPDCDE SGLFKAK	2895,3465	2895,488	-0,1415

	TQNDVDIADVAYYFEKDVK	2233,0659	2233,145	-0,0791
**Nestin**	EGWDPAVLASEGLEAPPSEK	2082,0025	2082,014	-0,0115
	EEVMEPPLEEESLEAKR	2014,9637	2014,947	0,0167
**Glypican 3**	YSQKAAR	823,4421	823,354	0,0881
	IWHFK	730,4035	730,386	0,0175

Mascot Score Histogram

Protein score is -10*Log(P), where P is the probability that the observed match is a random event.
Protein scores greater than 56 are significant ($p<0.05$).

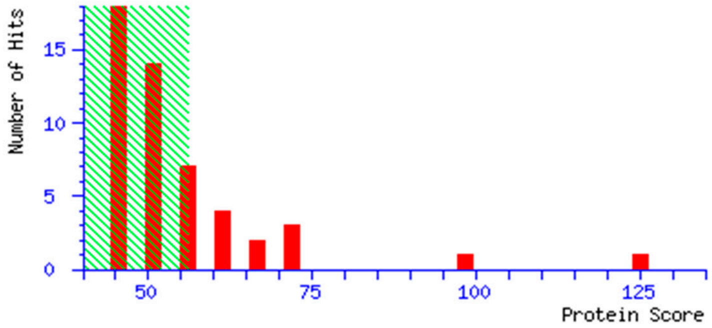

Supplementary Figure S6: Mascot Score Histogram. In the green area the proteins that were not reliably associated with the peptide list.
